# Electron‐Extraction Engineering Induced 1T’’‐1T’ Phase Transition of Re_0.75_V_0.25_Se_2_ for Ultrafast Sodium Ion Storage

**DOI:** 10.1002/advs.202205680

**Published:** 2022-11-13

**Authors:** Yuqiang Fang, Ximeng Lv, Zhuoran Lv, Yang Wang, Gengfeng Zheng, Fuqiang Huang

**Affiliations:** ^1^ State Key Laboratory of High‐Performance Ceramics and Superfine Microstructure Shanghai Institute of Ceramics Chinese Academy of Sciences Shanghai 200050 P. R. China; ^2^ Laboratory of Advanced Materials Department of Chemistry and Shanghai Key Laboratory of Molecular Catalysis and Innovative Materials Fudan University Shanghai 200438 P. R. China; ^3^ State Key Laboratory of Rare Earth Materials Chemistry and Applications College of Chemistry and Molecular Engineering Peking University Beijing 100871 P. R. China

**Keywords:** 1T’ Re_0.75_V_0.25_Se_2_, phase transition, ultrafast sodium‐ion storage

## Abstract

Inducing new phases of transition metal dichalcogenides by controlling the d‐electron‐count has attracted much interest due to their novel structures and physicochemical properties. 1T’’ ReSe_2_ is a promising candidate for sodium storage, but the low electronic conductivity and limited active sites hinder its electrochemical capacity. Herein, new‐phase 1T’ Re_0.75_V_0.25_Se_2_ crystals (P*2/m*) with zig‐zag chains are successfully synthesized. The 1T’’‐1T’ phase transition results from the electronic reorganization of 5d orbitals via electron extraction after V‐atom doping. The electrical conductivity of 1T’ Re_0.75_V_0.25_Se_2_ is 2.7 × 10^5^ times higher than that of 1T’’ ReSe_2_. Moreover, density functional theory (DFT) calculations reveal that 1T’ Re_0.75_V_0.25_Se_2_ has a larger interlayer spacing, lower bonding energy, and migration energy barrier for Na^+^ ions than 1T’’ ReSe_2_. As a result, 1T’ Re_0.75_V_0.25_Se_2_ electrode shows an excellent rate capability of 203 mAh g^−1^ at 50 C with no capacity fading over 5000 cycles for sodium storage, which is superior to most reported sodium‐ion anode materials. This 1T’ Re_0.75_V_0.25_Se_2_ provides a new platform for various applications such as electronics, catalysis, and energy storage.

## Graphical abstract

The phase transition of Re_1‐x_V_x_Se_2_ from 1T’’ to 1T’ phase can be achieved by decreasing the d‐electron count. The new‐phase 1T’ Re_0.75_V_0.25_Se_2_ has higher conductivity, larger interlayer distance, and lower migration energy barrier of Na^+^ ions, which results in an excellent rate capability of 203 mAh g^−1^ at 50 C with no capacity fading over 5000 cycles for sodium storage.

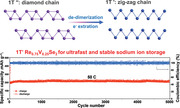

## 1 Introduction

Phase transition engineering of transition metal dichalcogenides (TMDs) has attracted much interest due to their unique electronic band structure and fascinating physicochemical properties.^[^ ^1^, ^2^, ^3^, ^4^, ^5^, ^6^, ^7^, ^8^ ^]^ Theocratically, the polymorphs of TMDs are strongly governed by the d‐electron count of transition metal atoms.^[^ ^4^, ^9^, ^10^, ^11^ ^]^ Phase transition of group 6 TMDs can occur from thermodynamically stable 2H to metastable phases (1T, 1T’, 1T’’’ , etc.) under chemically‐, thermally‐, strain‐, and electrostatic‐driven forces.^[^ ^12^, ^13^, ^14^, ^15^, ^16^, ^17^, ^18^, ^19^ ^]^ For instance, 2H MoS_2_ is transformed into 1T’/1T’’’ MoS_2_ through intercalation of alkali metal ions, because the injected electrons can make Mo atoms form various types of Mo—Mo bonding.^[^ ^20^, ^21^, ^22^ ^]^ Compared to the semiconducting 2H phase, 1T/1T’ phase shows metallic features, leading to better electrochemical performance and intriguing physics such as superconductivity and quantum spin Hall effect.^[^ ^23^, ^24^, ^25^, ^26^ ^]^

Different from group‐6 TMDs, Re*X*_2_ (*X* = S, Se) naturally adopts 1T’’ phase (*P*‐1) other than 2H phase. Owing to the Jahn–Teller effect, the extra unpaired electron of Re^4+^ makes the high‐energy t*_2g_* level further split to break the degeneracy, resulting in a large bandgap. ^[^ ^27^, ^28^, ^29^ ^]^ The adjacent four Re atoms are linked into the diamond‐shaped Re_4_ chains along the *b*‐axis. The well‐localized charge distribution makes the van der Waals (vdW) interaction weaker than other TMDs. Hence, 1T’’ ReSe_2_ facilitates the insertion/extraction of large radius Na^+^ as a promising anode for the sodium ions batteries.^[^ ^30^, ^31^, ^32^, ^33^, ^34^, ^35^, ^36^ ^]^ However, the volume expansion and low conductivity of 1T’’ ReSe_2_ still cannot match the dynamics of the high‐rate electrochemical reaction.^[^ ^37^ ^]^

To improve the electrochemical capacity of bulk ReSe_2_, phase transition engineering was carried out to optimize crystal structure and enhance electrical conductivity. Unlike the electron‐injection‐induced phase transition (2H‐1T/1T’), an electron‐extraction strategy was carried out to reduce electron localization of Re_4_ diamond chains in 1T’’ ReSe_2_, which would induce the formation of 1T’ phase with the Re_2_ zig‐zag chains.^[^ ^38^, ^39^ ^]^ Hence, V^4+^ (d^1^) is chosen to partially replace Re^4+^ (d^3^) of ReSe_2_, which will enhance the electron repulsion force and expand interlayer spacing. Moreover, the metallic Re_2_ zig‐zag chains can improve the electrical conductivity, thereby improving the performance of sodium ions storage.

Herein, we have successfully synthesized a new‐phase 1T’ Re_0.75_V_0.25_Se_2_ crystal and resolved its crystal structure through a single‐crystal X‐ray diffractor. The microstructural evolution from Re_4_ diamond chains (1T’’) to zig‐zag chains (1T’) was observed through a high‐angle annular dark‐field scanning transmission electron microscope (HAADF‐STEM). The electrochemical reaction process was detected via the in situ XRD. Compared to 1T’’ ReSe_2_, 1T’ Re_0.75_V_0.25_Se_2_ has some remarkable merits including better electrical conductivity, smaller Na^+^ binding energy, larger interlayer distance, and smaller migration energy barrier (DFT calculations). These features endow 1T’ Re_0.75_V_0.25_Se_2_ with excellent high‐rate capability (203 mAh g^−1^ at 50 C) with 100% capacity retention after 5000 cycles at 50 C, indicating no significant structural degradation. This phase transition strategy can guide the preparation of high‐performance ion‐storage materials.

## 2 Results and Discussion

**Figure 1:**
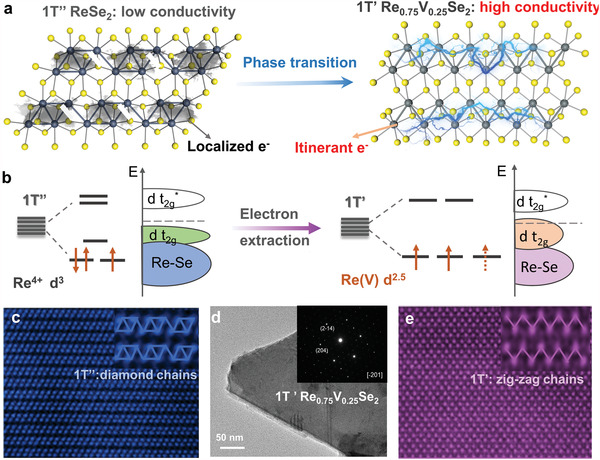
a) The atomically schematic diagram of phase transition from 1T’’ ReSe_2_ (diamond Re_4_ chains) to 1T’ Re_0.75_V_0.25_Se_2_ (zig‐zag chains) b) The splitting of the degenerate 5d orbitals of Re for 1T’’ and 1T’ phases using electron‐extraction engineering. c) Atomically resolved HAADF‐STEM images of the 1T’’ ReSe_2_ flake. d) Transmission electron microscope (TEM) image and SAED of 1T’ Re_0.75_V_0.25_Se_2_. e) HAADF‐STEM image of 1T’ Re_0.75_V_0.25_Se_2_ flake.

Chemical doping of heteroatoms is an effective method to induce the phase transition in TMDs family.^[^ ^2^, ^40^, ^41^, ^42^ ^]^ **Figure** **1**a shows the structural transition from 1T’’ ReSe_2_ to 1T’ Re_0.75_V_0.25_Se_2_ after V atom doping. In 1T’’ ReSe_2_, Re atoms deviate from the ideal octahedral sites, resulting in the formation of Re_4_ diamond chains along the *a*‐axis. When V^4+^ (*d* ^1^) substitutes Re^4+^ (*d* ^3^) of 1T’’ ReSe_2_, the electrons are extracted from the Re—Re d‐orbital, resulting in the de‐dimerization process from diamond Re—Re chains (d^3^) to zig‐zag chains (d^2.5^). Hence, the reorganization of 5d orbitals on electron extraction by V drives the phase transition (1T’’‐1T’) of ReSe_2_. As shown in Figure S1 (Supporting Information), the relative energy Δ*E* (1T’ vs 1T’’ Re_1‐x_V_x_Se_2_) decreases with increasing V content from 0 to 25%. The 1T’’ – 1T’ phase transition tendency reaches a maximum at *x* = 25% because of the lowest energy difference between the two phases. The decreased d‐electron count leads to the change of 5d orbital splitting modes. When the d‐electron count is 2.5, the electrons are filled in the degenerate *d_xy_*, *d_yz_*, and *d_xz_* levels (Figure 1b). Compared to the localized electron of Re_4_ diamond chains, electrons can move freely through the zig‐zag chains, resulting in the high conductivity of 1T’ Re_0.75_V_0.25_Se_2_.

Re_1‐x_V_x_Se_2_ (*x* = 0–0.25) alloy compounds were prepared through a high‐temperature solid‐state method. Figure S2 (Supporting Information) shows the typical lamellar shape of 1T’ Re_0.75_V_0.25_Se_2_ crystal. The V, Re, and Se elements are distributed uniformly in the energy‐dispersive X‐ray spectroscopy (EDS) elemental mapping, and the result reveals that the atomic ratio of V, Re, and Se is 0.26: 0.74: 2. (Table S1, Supporting Information) Moreover, the ICP‐AES results show that the atomic ratio of Re, V, and Se is 0.2458: 0.7542: 1.9745. To investigate the effect of doped V atoms in the structural evolution of 1T’’ ReSe_2_, the selected area electron diffraction (SAED) and HAADF‐STEM measurements were performed to analyze the atomic arrangements of 1T’’ ReSe_2_ and 1T’ Re_0.75_V_0.25_Se_2_. As shown in Figure 1d, the lattice plane (2‐14) and (204) of 1T’ Re_0.75_V_0.25_Se_2_ can be indexed. The diffraction spots of 1T’ ReSe_2_ flake can be indexed as (021) and (301) crystal planes (Figure S3, Supporting Information). Since the contrast of an atom depends directly on the atomic number in the HADF‐STEM image, hence Re atoms (Z = 73) stand out compared with V (Z = 23) and Se (Z = 36) atoms. As shown in Figure 1c, the Re_4_ diamond chains can be observed clearly in the STEM image for the basal plane of 1T’’ ReSe_2_. The substitutional V atoms are homogeneously distributed, Re/V atoms are linked by the zig‐zag chains, in agreement with the resolved crystal structure of 1T’ phase Re_0.75_V_0.25_Se_2_ (Figure 1e).

**Figure 2:**
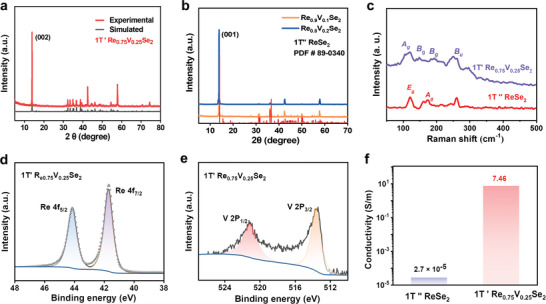
Structural charization and electrical properties of these two samples. a,b) The PXRD pattern of Re_1‐x_V_x_Se_2_ (*x* = 0.1, 0.2, 0.25) crystals. c) Raman spectra of 1T’’ ReSe_2_ and 1T’ Re_0.75_V_0.25_Se_2_ sample. d,e) High‐resolution Re 4*f* and V 2*p* XPS spectra of 1T’ Re_0.75_V_0.25_Se_2_ crystals. f) Electrical conductivity of 1T’’ ReSe_2_ and 1T’ Re_0.75_V_0.25_Se_2_.

Different from triclinic space group of 1T’’ ReSe_2_, 1T’ phase Re_0.75_V_0.25_Se_2_ crystallizes in a monoclinic space group P*2/m* (No. 11), with *a* = 5.85(6) Å, *b* = 3.30(3) Å, *c* = 12.82(14) Å, and *β* = 96.527(4)°. The powder X‐ray diffraction (XRD) pattern of Re_0.75_V_0.25_Se_2_ matches well with the simulated one from the single‐crystal structure of 1T’ Re_0.75_V_0.25_Se_2_ (**Figure** **2**a). The crystallographic parameters are seen in Tables S2–S5 (Supporting Information). Whereas the XRD patterns of Re_0.9_V_0.1_Se_2_ and Re_0.8_V_0.2_Se_2_ samples still reveal the phase of 1T’’ ReSe_2_ (Figure 2b). In addition, Raman spectroscopy was performed to detect the different vibration modes of these two structures. As shown in Figure 2c, the different Raman peaks of 1T’’ ReSe_2_ (120, 176, 212, 241, and 260 cm^−1^) and 1T’ Re_0.75_V_0.25_Se_2_ (117, 149, 195, 250, and 296 cm^−1^) indicate various vibration modes of Re—Re and Re—Se bonding, which derive from the renormalization of phonons after the doping of V atoms.

The element valence states were measured through X‐ray photoelectron spectroscopy (XPS). Figure 2d shows that the Re 4f_7/2_ and 4f_5/2_ peaks of 1T’ Re_0.75_V_0.25_Se_2_ sample are located at 41.7 and 44.1 eV, which are larger than those of 1T’’ ReSe_2_ with Re 4f_7/2_ = 41.3 eV and 4f_5/2_ = 43.8 eV.^[^ ^43^ ^]^ The higher binding energy of 1T’ Re_0.75_V_0.25_Se_2_ indicates that the doping of V atoms decreases the electronic density of the Re atoms. Moreover, the binding energy peaks at 521 and 513 eV corresponds to the V 2p_1/2_ and 2p_3/2_, which confirms the existence of doped V atoms (Figure 2e). The changed electronic density of Re atoms would affect the electrical conductivity. Figure 2f shows the electrical conductivity of 1T’’ ReSe_2_ and 1T’ Re_0.75_V_0.25_Se_2_, respectively. The conductivity value of 1T’ Re_0.75_V_0.25_Se_2_ is 7.46 S m^−1^, which is 2.7 × 10^5^ times that of 1T’’ ReSe_2_ (2.73 × 10^−5^ S m^−1^).

**Figure 3:**
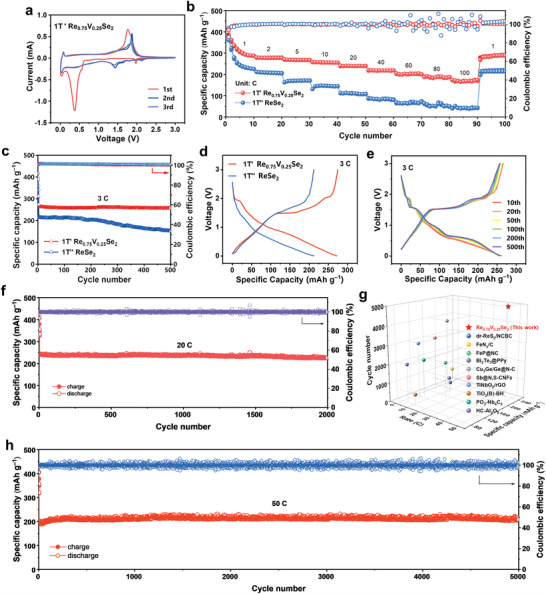
Electrochemical performance of 1T’ Re_0.75_V_0.25_Se_2_ electrode of SIBs. a) CV curves of 1T’ Re_0.75_V_0.25_Se_2_ for the initial three cycles at 0.2 mV s^−1^. b) Rate capacity of and 1T’’ ReSe_2_ c) Cycling performance at 3 C. d) GCD profiles of the two samples at 3 C. e) GCD profiles of 1T’ Re_0.75_V_0.25_Se_2_ at 3 C for different cycles. f) Long‐term cyclic performance of 1T’ Re_0.75_V_0.25_Se_2_ at 20 C for 2000 cycles. g) Comparison of the rate performance of our work with other reported anodes in SIBs. h) Cycling performance at 50 C for 5000 cycles.

Considering the improved conductivity of 1T’ Re_0.75_V_0.25_Se_2_, the sodium storage behavior of these two phases was evaluated by Na‐ion half cells. **Figure** **3**a shows the cyclic voltammogram (CV) curves of 1T’ Re_0.75_V_0.25_Se_2_ electrode for the initial three cycles at 0.2 mV s^−1^. At the first cathodic scan, the peak at 1.97 V can be ascribed to the initial sodiation process in 1T’ Re_0.75_V_0.25_Se_2_ to form nonstoichiometric Na^+^‐intercalated compounds Na_x_Re_0.75_V_0.25_Se_2_, and the peaks at 0.56 and 0.38 V represent the partial conversion from Na_x_Re_0.75_V_0.25_Se_2_ to Na_2_Se and metallic Re, as well as the formation of solid electrolyte interphase (SEI) layer. Another cathodic peak at nearly 0.01 V is related to the Na‐ions intercalation into carbon interlayers. Moreover, the peaks at 0.4 V shifted from 0.56 V correspond to the formation of NaVSe_2_, Na_2_Se, and metallic Re. For the anodic scan, the strong board peak at ≈1.77 V corresponds to the inverse processes of the above reactions. In the subsequent scans, two reversible cathodic/anodic pairs appear at ≈1.42–0.39 V and 1.36–1.85 V, respectively, indicating excellent reversibility. For comparison, the CV curves of 1T’’ ReSe_2_ show unsatisfied overlaps, indicating severe electrode degradation (Figure S4, Supporting Information).

Figure 3b presents the rate capabilities of 1T’ Re_0.75_V_0.25_Se_2_ and 1T’’ ReSe_2_ electrodes at different current densities. Remarkably, 1T’ Re_0.75_V_0.25_Se_2_ shows reversible capacities of 293, 275, 268, 258, 237, 222, 203, 184, and 173 mAh g^−1^ at current densities of 1 C, 2 C, 5 C, 10 C, 20 C, 40 C, 60 C, 80 C, 100 C, respectively, indicating higher capacities and rate capabilities compared to that of 1T’’ ReSe_2_. Figure 3c shows the cycling performances of 1T’ Re_0.75_V_0.25_Se_2_ and 1T’’ ReSe_2_ electrodes at 3 C. The 1T’ Re_0.75_V_0.25_Se_2_ electrode delivers initial charge/discharge capacities of 385.3/420.6 mAh g^−1^ at 1.5 C with the initial Coulomb efficiency (ICE) of 91.6%. The irreversible capacity is most likely caused by the formation of the SEI layer. Nonetheless, the CE quickly rises to 100% and the capacity remains stable after cycling. The 1T’ Re_0.75_V_0.25_Se_2_ electrode exhibits a reversible capacity of 260 mAh g^−1^ at 3 C with a capacity retention of 97% after 500 cycles. In comparison, the 1T’’ ReSe_2_ electrode suffers a relatively quick capacity fading after 200 cycles, retaining much less reversible capacity (151 mAh g^−1^) and inferior capacity retention (69%) after 500 cycles.

Figure 3d shows the galvanostatic charge/discharge (GCD) profiles of the two electrodes at 3 C, where the 1T’ Re_0.75_V_0.25_Se_2_ exhibits not only an elongated voltage platform at 1.5 V but also a much smaller polarization compared to 1T’’ ReSe_2_, proving its highly enhanced reversibility during sodiation/desodiation process. Selected GCD curves of 1T’ Re_0.75_V_0.25_Se_2_ electrode at 3 C show highly overlapped slopes at different cycles (10th, 20th, 50th, 100th, 200th, and 500th) (Figure 3e), indicating excellent reversibility of the charge/discharge process.

To further confirm the high‐rate and ultra‐stable sodium ion storage of 1T’ Re_0.75_V_0.25_Se_2_, the long‐term cycling performance of 1T’ Re_0.75_V_0.25_Se_2_ was carried out at high current densities. As shown in Figure S5 (Supporting Information), the 1T’ Re_0.75_V_0.25_Se_2_ can maintain the lamellar shape after charging and discharging process. The 1T’ Re_0.75_V_0.25_Se_2_ electrode shows reversible capacities of 225 and 203 mAh g^−1^ at 20 C and 50 C over 2000 and 5000 cycles (Figure 3f,h), respectively, which results from the highly stable crystal structure and large interlayer spacing of 1T’ Na_x_Re_0.75_V_0.25_Se_2_. In comparison with the reported sodium‐ion anodes (FeNx@C, FeP@NC, Bi_2_Te_3_@ppy, TiNbO_5_@rGO, etc.),^[^ ^44^, ^45^, ^46^, ^47^, ^48^, ^49^, ^50^, ^51^, ^52^, ^53^ ^]^ the 1T’ Re_0.75_V_0.25_Se_2_ electrode has very big advantages in ultra‐high rate capacity and long‐term cycling stability (Figure 3g).

**Figure 4:**
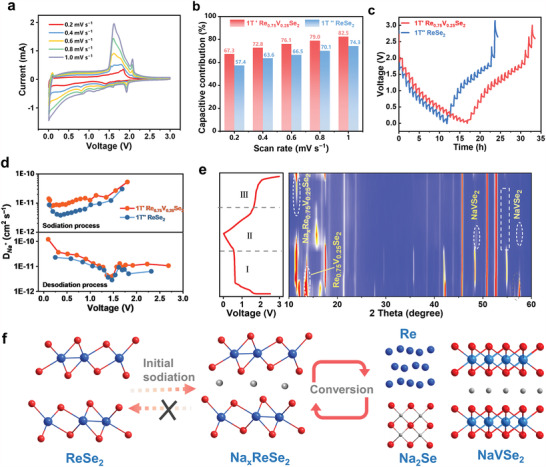
Electrochemical reaction kinetics analysis of 1T’ Re_0.75_V_0.25_Se_2_ electrode. a) CV curves at different scan rates for 1T’ Re_0.75_V_0.25_Se_2_ electrode from 0.2 to 1.0 mV s^−1^. b) Normalized capacitance contribution proportions at different scan rates. c) GITT potential profiles based on applied pulsed currents of 0.1 A g^−1^ for 10 min followed by relaxation intervals of 40 min. d) The diffusion coefficients of Na‐ion for 1T’ Re_0.75_V_0.25_Se_2_ versus 1T’’ ReSe_2_ electrodes calculated at different voltages during sodiation and desodiation process. e) In situ XRD of 1T’ Re_0.75_V_0.25_Se_2_ in the initial cycle. f) The diagram of the electrochemical reaction mechanism.

The reaction kinetic analysis based on CV curves was carried out at increasing sweep rates (0.2 to 1.0 mV s^−1^) to provide insight into the high‐rate and long‐life capabilities. As demonstrated in **Figure** **4**a, the pairs of cathodic and anodic peaks are well maintained with the increasing scan rate, suggesting a good reversible reaction in the 1T’ Re_0.75_V_0.25_Se_2_ electrode. Generally, the total charge transfer process can be divided into the faradaic reaction‐controlled and diffusion‐controlled process while the reaction‐controlled charge storage is intrinsically more quickly. To quantitatively determine the capacitive and diffusion‐controlled contributions, the total current density (*i*) at a fixed potential can be separated into two parts: *I* = *k*_1_ *v* + *k*_2_ *v* ^1/2^ where *k*_1_ and *k*_2_ are parameters and *k*_1_ *v* and *k*_2_ *v* ^1/2^ represent the capacitive and diffusion‐controlled processes, respectively. Figure S6 (Supporting Information) shows the separated capacitive contribution of 1T’ Re_0.75_V_0.25_Se_2_ and 1T’’ ReSe_2_ at 1.0 mV s^−1^. For 1T’ Re_0.75_V_0.25_Se_2_ electrode, the ratio gradually enlarged from 67.3% to 72.8%, 76.1%, 79.0%, and 82.5% as scan sweep rate increased (Figure 4b), presenting a higher fraction of the capacitive contribution at different scan rates compared to 1T’’ ReSe_2_. The enhanced capacitive storage points toward the origin of its high‐rate capabilities and long‐life stability, which is induced by the boosting of electrical conductivity and expansion of interlayer spacing.

The reaction resistance and Na‐ion diffusion coefficient (D_Na+_) of 1T’ Re_0.75_V_0.25_Se_2_ and 1T’’ ReSe_2_ electrodes are investigated by electrochemical impedance spectroscopy (EIS) and galvanostatic intermittent titration technique (GITT). Figure S7 (Supporting Information) shows the Nyquist plots of 1T’ Re_0.75_V_0.25_Se_2_ and 1T’’ ReSe_2_ electrodes after 10 cycles. Based on the radius of the semi‐circles, 1T’ Re_0.75_V_0.25_Se_2_ has lower charge transfer resistance than 1T’’ ReSe_2_. The GITT data are recorded by applying a series of pulsed currents (0.1 A g^−1^) for 10 min (pulse time, *τ*) followed by relaxation intervals of 40 min through the entire range of relevant voltages. Figure 4c presents the GITT curves of 1T’ Re_0.75_V_0.25_Se_2_ and 1T’’ ReSe_2_ electrodes. The D_Na+_ is calculated using the standard approach based on Fick's second law. The higher D_Na+_ values of the 1T’ Re_0.75_V_0.25_Se_2_ electrode reveal that the improved electrical conductivity and expansion of interlayer spacing can greatly facilitate Na‐ion transportation (Figure 4d).

To further understand the electrochemical reaction mechanism of the 1T’ Re_0.75_V_0.25_Se_2_ electrode, in situ XRD analyses were performed at its initial cycle (Figure 4e). At stage I, the 1T’ Re_0.75_V_0.25_Se_2_ XRD peak of (002) at 13.9° gradually shifts to lower angles, indicating the expanded interlayer spacing after the formation of intercalated compound Na_x_Re_0.75_V_0.25_Se_2_. At stage II, the peaks at 48.7° and 57.6° are indexed to the compound NaVSe_2_. At stage III, the characteristic peak of 1T’ Re_0.75_V_0.25_Se_2_ disappears, and the peak intensity of Na_x_Re_0.75_V_0.25_Se_2_ gradually increases, indicating the initial sodiation is an irreversible process. Figure 4f shows a schematic diagram of the sodium ion storage mechanism according to the following chemical equations:

(1) StageIInsertion:Re0.75V0.25Se2+Na++xe−→NaxRe0.75V0.25Se2

(2) StageIIConversion:NaxRe0.75V0.25Se2+3.25−xNa++3.25−xe−→1.5Na2Se+0.75Re+0.25NaVSe2

(3) StageIIIDesodiation:1.5Na2Se+0.75Re+0.25NaVSe2→NaxRe0.75V0.25Se2+3.25Na++3.25e−

Figure S8 (Supporting Information) shows the ex situ XPS spectra of Re and V elements at a fully discharged state and fully charged state. As shown in the Re 4f spectra, only metallic Re^0^ is detected in the full discharged state, indicating the complete reduction of Re^4+^. In addition, the peak of V 2p shifts to lower binding energy in the fully discharged sample, indicating the formation of the NaVSe_2_ intermediate. Afterward, in the fully charged states, the peaks of both Re 4f and V 2p shift to higher binding energy, approaching to their original states, which demonstrates the reversible sodium storage mechanism in cycling processes.

**Figure 5:**
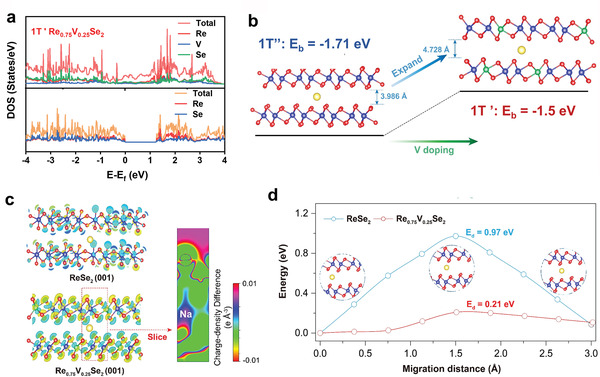
a) DOS calculations of 1T’’ ReSe_2_ and 1T’ Re_0.75_V_0.25_Se_2_. b) Difference in binding energy and interlayer distances of 1T’’ ReSe_2_ and 1T’ Re_0.75_V_0.25_Se_2_ after intercalation of Na^+^ ions. c) The charge density difference and corresponding 2D slice. d) The migration energy barriers in 1T’’ ReSe_2_ and 1T’ Re_0.75_V_0.25_Se_2_.

Density functional theory (DFT) calculations were performed to understand the better high‐rate sodium storage performance of 1T’ Re_0.75_V_0.25_Se_2_ than 1T’’ ReSe_2_. **Figure** **5**a shows the calculated density of states (DOS) for 1T’’ ReSe_2_ and 1T’ Re_0.75_V_0.25_Se_2_, respectively. The bandgap (*E*_g_) of 1T’’ ReSe_2_ is evaluated to be ≈1.18 eV, while no clear band gap was found for 1T’ Re_0.75_V_0.25_Se_2_, suggesting a better electronic migration capacity. To further compare the difference in Na^+^ storage kinetics between these two phases, the binding energy (*E*_b_) values are calculated to be  −1.71 and −1.50 eV for 1T’’ ReSe_2_ and 1T’ Re_0.75_V_0.25_Se_2_ system, respectively, with the optimized slab models of ReSe_2_ (001) and 1T’ Re_0.75_V_0.25_Se_2_ (001) (Figure 5b). In addition, the interlayer distance of 1T’ Re_0.75_V_0.25_Se_2_ is ≈19% larger than that of 1T’’ ReSe_2_ (4.728 Å vs 3.986 Å), which facilitates the migration of Na^+^ in the charge/discharge process.

The charge‐density difference and corresponding 2D slice depict the electronic transfer states after the insertion of Na^+^ ions (Figure 5c). It can be observed that the charge density of 1T’ Re_0.25_V_0.75_Se_2_ changes more than that of 1T’’ ReSe_2_ after Na^+^ ion intercalation. Also, the charge transfer (≈0.005 e Å^−3^) of 1T’ Na_1_Re_0.25_V_0.75_Se_2_ implies a delocalized state of Na^+^, which is beneficial to improving Na^+^ storage capacity. Most importantly, 1T’ Re_0.75_V_0.25_Se_2_ has a significantly lower energy barrier for Na^+^ ion diffusion (*E*_d_) based on the kinetics assessments with the climbing image nudged elastic band (CINEB) method. As shown in Figure 5d, the migration energy barrier *E*_d_ for 1T’ Re_0.75_V_0.25_Se_2_ (≈0.21 eV) is dramatically reduced than that of 1T’’ ReSe_2_ (≈0.97 eV). Overall, our theoretical calculations reveal the mechanism of the enhanced Na^+^ storage performance of 1T’ Re_0.75_V_0.25_Se_2_ compared to 1T’’ ReSe_2_.

## 3 Conclusion

In summary, we have successfully induced a new 1T’ phase of ReSe_2_ by V atoms doping. 1T’ Re_0.75_V_0.25_Se_2_ has typical Re—Re zig‐zag chains, different from the Re_4_ diamond chains of 1T’’ ReSe_2_. The doped V atoms give rise to enhancement of the conductivity and interlayer spacing, at the same time, the binding energy and migration barriers of Na^+^ are decreased a lot. Therefore, the high conductivity enables fast charge transfer and accelerated reaction kinetics. The weak van der Waals interaction and low migration barrier facilitate fast ion diffusion, resulting in high structural stability at high‐rate measurements. 1T’ Re_0.75_V_0.25_Se_2_ has a reversible capacity of 173 mAh g^−1^ at 100 C, which is 4.3 times that of 1T’’ ReSe_2_ (40 mAh g^−1^ at 100 C). In addition, the capacity of 1T’ Re_0.75_V_0.25_ shows 203 mAh g^−1^ (50 C) with no decay after 5000 cycles, indicating a high long‐term cyclability. This work provides a promising strategy for inducing new phases and improving the Na^+^ ions storage performance of TMDs materials.

## Conflict of Interest

The authors declare no conflict of interest.

## Supporting information

Supporting Information

## Data Availability

The data that support the findings of this study are available from the corresponding author upon reasonable request.

## References

[1] K. Hernandez Ruiz , Z. Wang , M. Ciprian , M. Zhu , R. Tu , L. Zhang , W. Luo , Y. Fan , W. Jiang , Small Sci. 2022, 2, 2100047. 10.1002/smsc.202100047 PMC11935802 40212328

[2] V. Kochat , A. Apte , J. A. Hachtel , H. Kumazoe , A. Krishnamoorthy , S. Susarla , J. C. Idrobo , F. Shimojo , P. Vashishta , R. Kalia , A. Nakano , C. S. Tiwary , P. M. Ajayan , Adv. Mater. 2017, 29, 1703754. 10.1002/adma.201703754 28990227

[3] T. Rao , H. Wang , Y.‐J. Zeng , Z. Guo , H. Zhang , W. Liao , Adv. Sci. 2021, 8, 2002284. 10.1002/advs.202002284 PMC8132069 34026429

[4] D. Voiry , A. Goswami , R. Kappera , C. d. C. C. e Silva , D. Kaplan , T. Fujita , M. Chen , T. Asefa , M. Chhowalla , Nat. Chem. 2015, 7, 45. 25515889 10.1038/nchem.2108

[5] R. Wang , Y. Yu , S. Zhou , H. Li , H. Wong , Z. Luo , L. Gan , T. Zhai , Adv. Funct. Mater. 2018, 28, 1802473.

[6] X. Wang , Z. Song , W. Wen , H. Liu , J. Wu , C. Dang , M. Hossain , M. A. Iqbal , L. Xie , Adv. Mater. 2019, 31, 1804682. 10.1002/adma.201804682 30393917

[7] H. Yang , S. W. Kim , M. Chhowalla , Y. H. Lee , Nat. Phys. 2017, 13, 931.

[8] W. Zhao , J. Pan , Y. Fang , X. Che , D. Wang , K. Bu , F. Huang , Chem. – A Euro. J. 2018, 24, 15942. 10.1002/chem.201801018 29693734

[9] W. Li , X. Qian , J. Li , Nat. Rev. Mater. 2021, 6, 829.

[10] Z. Sun , H. Lv , Z. Zhuo , A. Jalil , W. Zhang , X. Wu , J. Yang , J. Mater. Chem. C 2018, 6, 1248.

[11] Q. H. Wang , K. Kalantar‐Zadeh , A. Kis , J. N. Coleman , M. S. Strano , Nat. Nanotechnol. 2012, 7, 699. 23132225 10.1038/nnano.2012.193

[12] K.‐A. N. Duerloo , Y. Li , E. J. Reed , Nat. Commun. 2014, 5, 4214. 24981779 10.1038/ncomms5214

[13] Y. Fang , X. Hu , W. Zhao , J. Pan , D. Wang , K. Bu , Y. Mao , S. Chu , P. Liu , T. Zhai , F. Huang , J. Am. Chem. Soc. 2019, 141, 790. 30601005 10.1021/jacs.8b12133

[14] R. A. Gordon , D. Yang , E. D. Crozier , D. T. Jiang , R. F. Frindt , Phys. Rev. B 2002, 65, 125407.

[15] W. Hou , A. Azizimanesh , A. Sewaket , T. Peña , C. Watson , M. Liu , H. Askari , S. M. Wu , Nat. Nanotechnol. 2019, 14, 668. 31182837 10.1038/s41565-019-0466-2

[16] J. Peng , Y. Liu , X. Luo , J. Wu , Y. Lin , Y. Guo , J. Zhao , X. Wu , C. Wu , Y. Xie , Adv. Mater. 2019, 31, 1900568. 10.1002/adma.201900568 30920692

[17] S. Song , D. H. Keum , S. Cho , D. Perello , Y. Kim , Y. H. Lee , Nano Lett. 2016, 16, 188. 26713902 10.1021/acs.nanolett.5b03481

[18] F. Zhang , H. Zhang , S. Krylyuk , C. A. Milligan , Y. Zhu , D. Y. Zemlyanov , L. A. Bendersky , B. P. Burton , A. V. Davydov , J. Appenzeller , Nat. Mater. 2019, 18, 55. 30542093 10.1038/s41563-018-0234-y

[19] G.‐H. Nam , Q. He , X. Wang , Y. Yu , J. Chen , K. Zhang , Z. Yang , D. Hu , Z. Lai , B. Li , Q. Xiong , Q. Zhang , L. Gu , H. Zhang , Adv. Mater. 2019, 31, 1807764. 10.1002/adma.201807764 30972852

[20] C. Shang , Y. Q. Fang , Q. Zhang , N. Z. Wang , Y. F. Wang , Z. Liu , B. Lei , F. B. Meng , L. K. Ma , T. Wu , Z. F. Wang , C. G. Zeng , F. Q. Huang , Z. Sun , X. H. Chen , Phys. Rev. B 2018, 98, 184513.

[21] Z. Lai , Q. He , T. H. Tran , D. V. M. Repaka , D.‐D. Zhou , Y. Sun , S. Xi , Y. Li , A. Chaturvedi , C. Tan , B. Chen , G.‐H. Nam , B. Li , C. Ling , W. Zhai , Z. Shi , D. Hu , V. Sharma , Z. Hu , Y. Chen , Z. Zhang , Y. Yu , X. Renshaw Wang , R. V. Ramanujan , Y. Ma , K. Hippalgaonkar , H. Zhang , Nat. Mater. 2021, 20, 1113. 33859384 10.1038/s41563-021-00971-y

[22] C. Guo , J. Pan , H. Li , T. Lin , P. Liu , C. Song , D. Wang , G. Mu , X. Lai , H. Zhang , W. Zhou , M. Chen , F. Huang , J. Mater. Chem. C 2017, 5, 10855.

[23] M. A. Lukowski , A. S. Daniel , F. Meng , A. Forticaux , L. Li , S. Jin , J. Am. Chem. Soc. 2013, 135, 10274. 23790049 10.1021/ja404523s

[24] Q. Tang , D. Jiang , ACS Catal. 2016, 6, 4953.

[25] P. Chen , W. W. Pai , Y.‐H. Chan , W.‐L. Sun , C.‐Z. Xu , D.‐S. Lin , M. Y. Chou , A.‐V. Fedorov , T.‐C. Chiang , Nat. Commun. 2018, 9, 2003. 29784909 10.1038/s41467-018-04395-2 PMC5962594

[26] S. Tang , C. Zhang , D. Wong , Z. Pedramrazi , H.‐Z. Tsai , C. Jia , B. Moritz , M. Claassen , H. Ryu , S. Kahn , J. Jiang , H. Yan , M. Hashimoto , D. Lu , R. G. Moore , C.‐C. Hwang , C. Hwang , Z. Hussain , Y. Chen , M. M. Ugeda , Z. Liu , X. Xie , T. P. Devereaux , M. F. Crommie , S.‐K. Mo , Z.‐X. Shen , Nat. Phys. 2017, 13, 683.

[27] X. Li , C. Chen , Y. Yang , Z. Lei , H. Xu , Adv. Science 2020, 7, 2002320. 10.1002/advs.202002320 PMC7709994 33304762

[28] J. V. Marzik , R. Kershaw , K. Dwight , A. Wold , J. Solid State Chem. 1984, 51, 170.

[29] M. Rahman , K. Davey , S.‐Z. Qiao , Adv. Funct. Mater. 2017, 27, 1606129.

[30] Y. Liao , C. Chen , D. Yin , Y. Cai , R. He , M. Zhang , Nano‐Micro Lett. 2019, 11, 22. 10.1007/s40820-019-0248-2 PMC7770771 34137959

[31] Z. Xia , X. Chen , H. Ci , Z. Fan , Y. Yi , W. Yin , N. Wei , J. Cai , Y. Zhang , J. Sun , J. Energy Chem 2021, 53, 155.

[32] J. Zhou , Y. Zhang , Z. Liu , Z. Qiu , D. Wang , Q. Zeng , C. Yang , K. N. Hui , Y. Yang , Z. Peng , S. Guo , Sci. China Mater 2022, 40843. 10.1007/s40843-022-2073-y

[33] Q. Zhou , D. Wang , L. Ni , H. Zhang , J. Zhao , J. Alloys Compd. 2022, 909, 164773.

[34] M. Zhuang , G.‐L. Xu , L.‐Y. Gan , Y. Dou , C.‐J. Sun , X. Ou , Y. Xie , Z. Liu , Y. Cai , Y. Ding , I. H. Abidi , A. Tyagi , K. Amine , Z. Luo , Nano Energy 2019, 58, 660.

[35] W.‐C. Lin , Y.‐C. Yang , H.‐Y. Tuan , Energy Storage Mater. 2022, 51, 38.

[36] M. Wu , J. Yang , D. H. L. Ng , J. Ma , physica status solidi RRL 2019, 13, 1900329.

[37] W. Zong , H. Guo , Y. Ouyang , L. Mo , C. Zhou , G. Chao , J. Hofkens , Y. Xu , W. Wang , Y.‐E. Miao , G. He , I. P. Parkin , F. Lai , T. Liu , Adv. Funct. Mater. 2022, 32, 2110016.

[38] Y.‐C. Kao , T. Huang , D.‐Y. Lin , Y.‐S. Huang , K.‐K. Tiong , H.‐Y. Lee , J.‐M. Lin , H.‐S. Sheu , C.‐M. Lin , J. Chem. Phys. 2012, 137, 024509. 22803549 10.1063/1.4733985

[39] I. H. Kwak , I. S. Kwon , T. T. Debela , H. G. Abbas , Y. C. Park , J. Seo , J.‐P. Ahn , J. H. Lee , J. Park , H. S. Kang , ACS Nano 2020, 14, 11995. 32813497 10.1021/acsnano.0c05159

[40] R. Dahal , L. Z. Deng , N. Poudel , M. Gooch , Z. Wu , H. C. Wu , H. D. Yang , C. K. Chang , C. W. Chu , Phys. Rev. B 2020, 101, 140505.

[41] P. Li , J. Cui , J. Zhou , D. Guo , Z. Zhao , J. Yi , J. Fan , Z. Ji , X. Jing , F. Qu , C. Yang , L. Lu , J. Lin , Z. Liu , G. Liu , Adv. Mater. 2019, 31, 1904641. 10.1002/adma.201904641 31595592

[42] L. Sun , M. Gao , Z. Jing , Z. Cheng , D. Zheng , H. Xu , Q. Zhou , J. Lin , Chem. Engineer. J. 2022, 429, 132187.

[43] Z. Lai , A. Chaturvedi , Y. Wang , T. H. Tran , X. Liu , C. Tan , Z. Luo , B. Chen , Y. Huang , G.‐H. Nam , Z. Zhang , Y. Chen , Z. Hu , B. Li , S. Xi , Q. Zhang , Y. Zong , L. Gu , C. Kloc , Y. Du , H. Zhang , J. Am. Chem. Soc. 2018, 140, 8563. 29870234 10.1021/jacs.8b04513

[44] S. Liu , K. Niu , S. Chen , X. Sun , L. Liu , B. Jiang , L. Chu , X. Lv , M. Li , Carbon Energy 2022, 4, 645.

[45] X. Liu , Y. Si , K. Li , Y. Xu , Z. Zhao , C. Li , Y. Fu , D. Li , Energy Storage Mater. 2021, 41, 255.

[46] Z. Liu , H. Sun , X. Wang , Z.‐Y. Gu , C. Xu , H. Li , G. Zhang , Y. He , X.‐L. Wu , Energy Storage Mater. 2022, 48, 90.

[47] X. Lu , Y. Shi , D. Tang , X. Lu , Z. Wang , N. Sakai , Y. Ebina , T. Taniguchi , R. Ma , T. Sasaki , C. Yan , ACS Nano 2022, 16, 4775. 35235304 10.1021/acsnano.2c00089

[48] C. Shang , L. Hu , D. Luo , K. Kempa , Y. Zhang , G. Zhou , X. Wang , Z. Chen , Adv. Sci. 2020, 7, 2002358. 10.1002/advs.202002358 PMC7675052 33240776

[49] B. Sun , Q. Lu , K. Chen , W. Zheng , Z. Liao , N. Lopatik , D. Li , M. Hantusch , S. Zhou , H. I. Wang , Z. Sofer , E. Brunner , E. Zschech , M. Bonn , R. Dronskowski , D. Mikhailova , Q. Liu , D. Zhang , M. Yu , X. Feng , Adv. Mater. 2022, 34, 2108682. 10.1002/adma.202108682 35148441

[50] C. Wang , J. Yan , T. Li , Z. Lv , X. Hou , Y. Tang , H. Zhang , Q. Zheng , X. Li , Angew. Chem. Inter. Ed. 2021, 60, 25013. 10.1002/anie.202110177 34523206

[51] W. Zong , C. Yang , L. Mo , Y. Ouyang , H. Guo , L. Ge , Y.‐E. Miao , D. Rao , J. Zhang , F. Lai , T. Liu , Nano Energy 2020, 77, 105189.

[52] C. Yu , Y. Li , H. Ren , J. Qian , S. Wang , X. Feng , M. Liu , Y. Bai , C. Wu , Carbon Energy 2022, 10.1002/cey2.220.

[53] H. Xia , L. Zan , G. Qu , Y. Tu , H. Dong , Y. Wei , K. Zhu , Y. Yu , Y. Hu , D. Deng , J. Zhang , Energy Environ. Sci. 2022, 15, 771.

