# Supplementary material for: Electron‐Extraction Engineering Induced 1T’’‐1T’ Phase Transition of Re0.75V0.25Se2 for Ultrafast Sodium Ion Storage
Source: Adv Sci (Weinh). 2022 Nov 13;9(36):2205680. doi: 10.1002/advs.202205680 (PMC9798975; doi:10.1002/advs.202205680)
Supplement: Supplementary file 1 — Supporting Information [file ADVS-9-2205680-s001.pdf]

ADVANCED SCIENCE
Open Access

Supporting Information

for *Adv. Sci.*, DOI 10.1002/advs.202205680

Electron-Extraction Engineering Induced 1T''-1T' Phase Transition of $Re_{0.75}V_{0.25}Se_2$ for Ultrafast Sodium Ion Storage

Yuqiang Fang, Ximeng Lv, Zhuoran Lv, Yang Wang, Gengfeng Zheng and Fuqiang Huang**

Supporting Information

Electron-Extraction-Engineering Induced 1T”-1T’ phase Transition of $Re_{0.75}V_{0.25}Se_2$ for Ultrafast Sodium Ion Storage

Yuqiang Fang[#], Mengxi Lv[#], Zhuoran Lv[#], Yang Wang, Gengfeng Zheng[*], Fuqiang Huang[*]

Experimental Section

Sample Preparation. $Re_{1-x}V_xSe_2$ (x = 0, 0.1, 0.2, 0.25) powders were synthesized through a high-temperature solid-state reaction. The reactants Re, V, and Se powders were mixed in the stoichiometric moral ratio and sealed in evacuated silica tubes. These tubes were heated up to 1200 °C at 5 °C/min and then kept for 24h. The $Re_{1-x}V_xSe_2$ polycrystalline samples are shiny and gray.

Structure characterization. The single-crystal X-ray diffraction of $Re_{0.75}V_{0.25}Se_2$ crystal was performed in a Bruker D8QUEST diffractometer with Mo Kα (λ = 0.71073 Å). The powder X-ray diffraction of the $Re_{1-x}V_xSe_2$ sample was detected using a Bruker D8QUEST diffractometer with Cu-Kα target ranging from 5° to 80°. The Raman spectra were measured in a HORIBA Raman spectrometer. The atomic microstructures of $ReSe_2$ and $Re_{0.75}V_{0.25}Se_2$ were observed through scanning transmission electron microscopy (aberration-corrected Nion HERMES-100).

Electrochemical Characterization. Electrochemical measurements were carried out by using CR2032 coin cells. The working electrode slurry was prepared by mixing active materials ($ReSe_2$, $Re_{0.75}V_{0.25}Se_2$), conductive carbon black (Super P), and sodium carboxyl-methyl cellulose (CMC-Na) in a mass ratio of 8:1:1 with water as the solvent. Then the slurry was coated on a copper foil and dried under vacuum at 80° C for 12h. After that, the foil was punched into 12 mm discs. The average mass loading of active material on each disc was ≈1.2 mg cm^{-2}. The half cells were assembled with fresh sodium foil as the counter electrode and glass fiber (GF/D, Whatman) as a separator in an argon-filled glove box (MBRAUN, <0.5 ppm of both oxygen and water). The electrolyte was 1.0 M $NaPF_6$ dissolved in DIGLYME (NP-005). The charge-discharge tests were carried out on LAND-CT2001A test system at different current densities. Cyclic voltammogram (CV) from 0.01 to 3.0 V and electrochemical impedance spectroscopy (EIS) over the frequency from 100 kHz to 0.1 Hz were measured by CHI760E electrochemical workstation (Chenhua).

Density functional theory Calculation. Density functional theory (DFT) calculations were performed with the Vienna *ab initio* simulation package (VASP), using the plane-wave basis with an energy cutoff of 600 eV,[1-2] projectors augmented wave

(PAW) potentials,[3] and the generalized gradient approximation (GGA) with the Perdew−Burke−Ernzerhof (PBE) exchange-correlation functional.[4] All structures were relaxed by a conjugate gradient (CG) method until the residual force component was less than 0.03 eV/Å, and the convergence criterion of total energy in the self-consistent field method was set to 10^{-6} eV. The vacuum space was set to be 20 Å to separate the interactions between the neighboring slabs. In this calculation, a 1 × 4 × 1 k-point mesh was adopted for $ReSe_2$ (4 × 4 × 1) supercell, and 1 × 8 × 1 mesh for $Re_{0.75}V_{0.25}Se_2$ (4 × 4 × 1) supercell. Climbing image-nudged elastic band (CINEB) method [42] was also employed for sodium ion migration calculations.

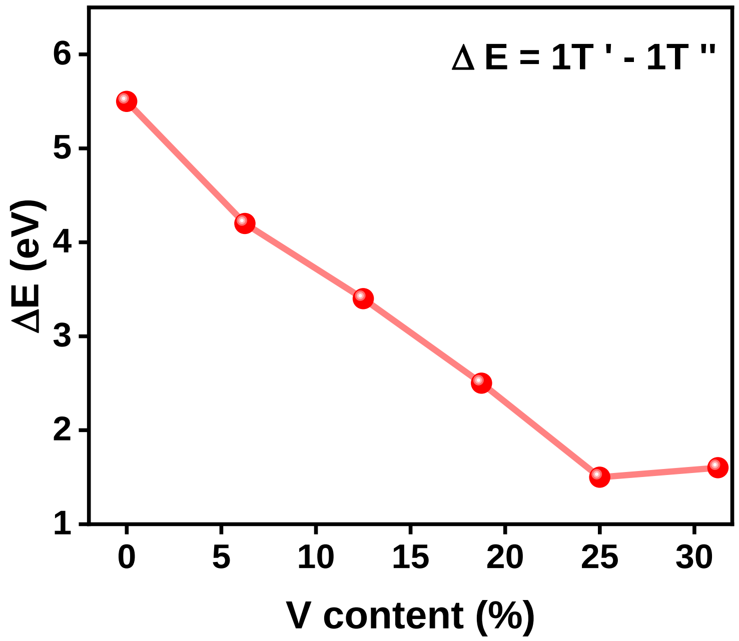

Figure S1. Relative energy per supercell of 1T' $Re_{1-x}V_xSe_2$ compared to 1T'' $Re_{1-x}V_xSe_2$.

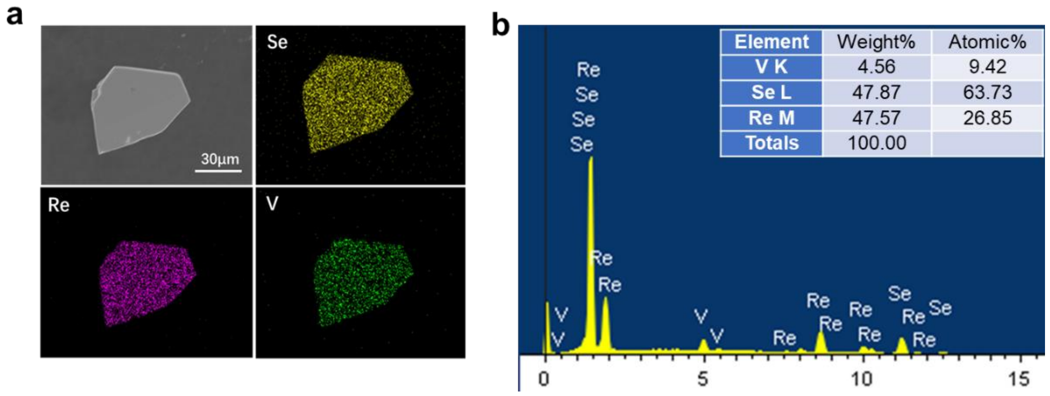

Figure S2. a) SEM image and EDS element mapping of Re, V and Se atoms for 1T' Re0.75V0.25Se2 crystal. b) EDS result of 1T' $Re_{0.75}V_{0.25}Se_2$ crystal.

Table S1. EDS results of various $Re_{1-x}V_xSe_2$ crystals

$Re_{1-x}V_xSe_2$	Exact ratio	Re (%)	V (%)	Se (%)
X=0.1	0.091	32.81	3.28	63.91
X=0.2	0.207	28.71	7.49	63.8
X=0.25	0.259	26.85	9.42	63.73

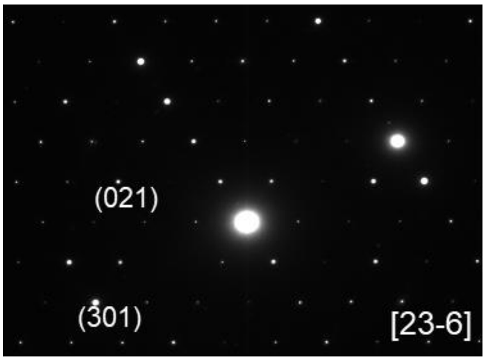

Figure S3. Selected area electron diffraction (SAED) and 1T'' $ReSe_2$ flake.

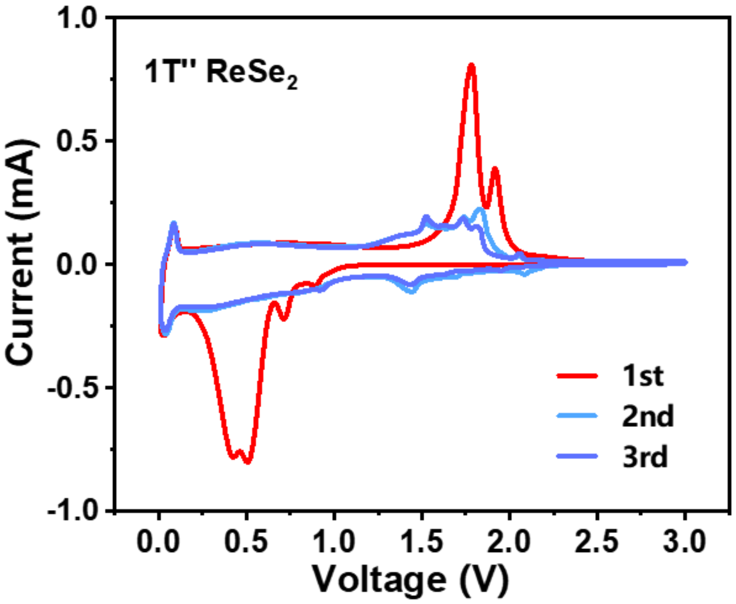

Figure S4. CV curves of 1T’ $Re_{0.75}V_{0.25}Se_2$

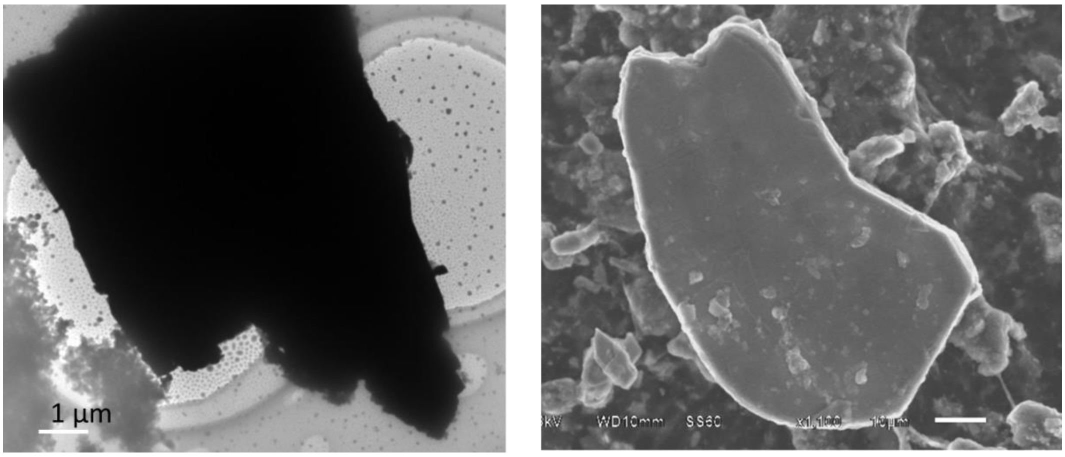

Figure S5. Low-migration TEM and SEM images of sample during charging and discharging.

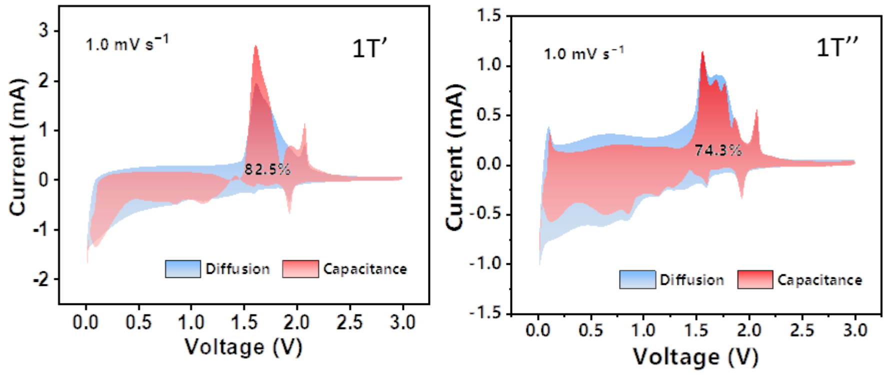

Figure S6. The separated capacitive contribution of 1T’ $Re_{0.75}V_{0.25}Se_2$ and 1T’’ $ReSe_2$ at 1.0 mV s^{-1}

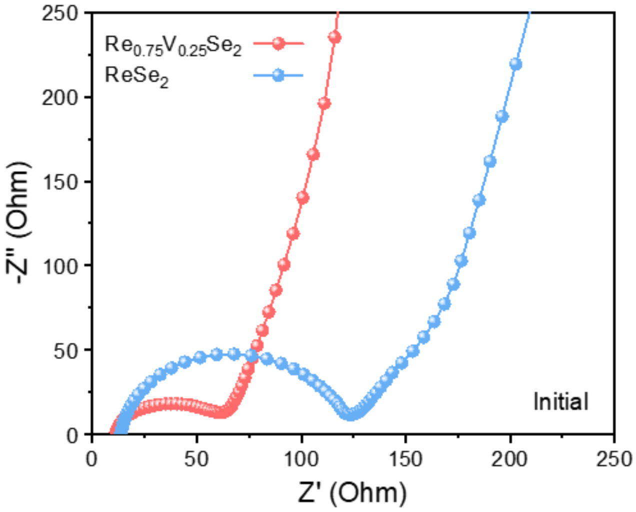

Figure S7. Nyquist plots of 1T’ $Re_{0.75}V_{0.25}Se_2$ and 1T’’ $ReSe_2$

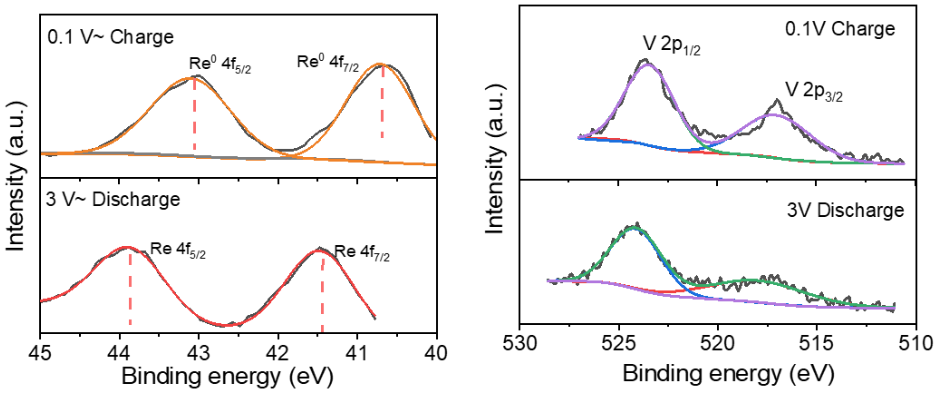

Figure S8. Ex-situ XPS spectra of Re 4f and V 2p tested at discharge-charge states.

Table S2. Crystallographic data and details of the structure refinements of 1T' $Re_{0.75}V_{0.25}Se_2$

Chemical formula	$Re_{0.75}$ $V_{0.25}Se_2$
M_r	317.53
Crystal system, space group	Monoclinic, $P2_1/m$
Temperature (K)	273
a, b, c (Å)	5.8576 (6), 3.3000 (3), 12.8217 (14)
β (°)	96.527 (4)
V (Å^3)	246.24 (4)
Z	4
Radiation type	Mo $K\alpha$
μ (mm^{-1})	69.58
Diffractometer	CCD area detector
Absorption correction	–
No. of measured, independent and observed [$I > 2\sigma(I)$] reflections	3556, 505, 411
R_{int}	0.058
$(\sin \theta/\lambda)_{max}$ (Å^{-1})	0.594
$R[F^2 > 2\sigma(F^2)]$, $wR(F^2)$, S	0.037, 0.102, 1.05
No. of reflections	505
No. of parameters	39
$\Delta\rangle_{max}$, $\Delta\rangle_{min}$ (e Å^{-3})	2.25, -1.74

Table S3. Fractional atomic coordinates and isotropic or equivalent isotropic displacement parameters (Å^2) for 1T' $Re_{0.75}V_{0.25}Se_2$

	x	y	z	U_{iso}*/U_{eq}
Re01	0.29993 (16)	0.750000	0.49656 (7)	0.0247 (5)
V01	0.29993 (16)	0.750000	0.49656 (7)	0.0247 (5)
Re02	0.80157 (16)	0.750000	1.00271 (7)	0.0243 (5)
V02	0.80157 (16)	0.750000	1.00271 (7)	0.0243 (5)
Se03	0.5523 (3)	0.250000	0.89238 (15)	0.0136 (5)
Se04	0.1069 (3)	0.250000	0.60789 (15)	0.0143 (6)
Se05	0.6136 (3)	0.750000	0.63859 (15)	0.0160 (6)
Se06	1.0479 (3)	0.750000	0.86142 (16)	0.0164 (6)

Table S4. Atomic displacement parameters (Å^2) for 1T' $Re_{0.75}V_{0.25}Se_2$

	U^{11}	U^{22}	U^{33}	U^{12}	U^{13}	U^{23}
Re01	0.0072 (6)	0.0559 (9)	0.0110 (7)	0.000	0.0008 (4)	0.000
V01	0.0072 (6)	0.0559 (9)	0.0110 (7)	0.000	0.0008 (4)	0.000
Re02	0.0067 (6)	0.0564 (8)	0.0104 (6)	0.000	0.0034 (4)	0.000
V02	0.0067 (6)	0.0564 (8)	0.0104 (6)	0.000	0.0034 (4)	0.000
Se03	0.0098 (10)	0.0128 (1)	0.0197 (11)	0.000	0.0082 (7)	0.000
Se04	0.0081 (10)	0.0110 (1)	0.0226 (12)	0.000	-0.0028 (7)	0.000
Se05	0.0139 (11)	0.0096 (1)	0.0226 (12)	0.000	-0.0057 (7)	0.000
Se06	0.0164 (11)	0.0111 (11)	0.0243 (12)	0.000	0.0140 (8)	0.000

Table S5. Selected bond distances (Å) and bond angles (^{o}) of 1T' $Re_{0.75}V_{0.25}Se_2$

Bond distances		Bond distances	
Re1—Se5	2.435 (2)	Re2—Se6	2.4408 (19)
Re1—Se5^{i}	2.4862 (16)	Re2—Se6^{v}	2.4871 (17)
Re1—Se5^{ii}	2.4862 (16)	Re2—Se6^{vi}	2.4871 (17)
Re1—Se4	2.5310 (15)	Re2—Se3^{iii}	2.5272 (15)
Re1—Se4^{iii}	2.5311 (15)	Re2—Se3	2.5272 (16)
Re1—Se4^{iv}	2.597 (2)	Re2—Se3^{vii}	2.5959 (19)
Re1—Re1^{ii}	2.8596 (15)	Re2—Re2^{v}	2.8580 (15)
Re1—Re1^{i}	2.8596 (15)	Re2—Re2^{vi}	2.8580 (15)
Bond angles		Bond angles	
Se5—Re1—Se5^{i}	108.97 (6)	Se6—Re2—Se3	86.40 (6)
Se5—Re1—Se5^{ii}	108.97 (6)	Se6^{v}—Re2—Se3	164.06 (7)
Se5^{i}—Re1—Se5^{ii}	83.16 (7)	Se6^{vi}—Re2—Se3	95.47 (5)
Se5—Re1—Se4	85.75 (6)	Se3^{iii}—Re2—Se3	81.52 (6)
Se5^{i}—Re1—Se4	95.73 (5)	Se6—Re2—Se3^{vii}	163.48 (8)
Se5^{ii}—Re1—Se4	164.84 (7)	Se6^{v}—Re2—Se3^{vii}	82.96 (6)
Se5—Re1—Se4^{iii}	85.74 (6)	Se6^{vi}—Re2—Se3^{vii}	82.96 (6)
Se5^{i}—Re1—Se4^{iii}	164.84 (7)	Se3^{iii}—Re2—Se3^{vii}	81.11 (6)
Se5^{ii}—Re1—Se4^{iii}	95.73 (5)	Se3—Re2—Se3^{vii}	81.11 (6)
Se4—Re1—Se4^{iii}	81.37 (6)	Se6—Re2—Re2^{v}	55.31 (5)
Se5—Re1—Se4^{iv}	162.84 (8)	Se6^{v}—Re2—Re2^{v}	53.80 (4)
Se5^{i}—Re1—Se4^{iv}	83.58 (6)	Se6^{vi}—Re2—Re2^{v}	100.10 (6)
Se5^{ii}—Re1—	83.58 (6)	Se3^{iii}—Re2—	91.68 (4)

Se4^{iv}		Re2^{v}	
Se4—Re1—Se4^{iv}	81.27 (6)	Se03—Re02—Re02^{v}	141.55 (7)
Se4^{iii}—Re1—Se4^{iv}	81.27 (6)	Se3^{vii}—Re2—Re2^{v}	135.41 (4)
Se5—Re1—Re1^{ii}	55.31 (4)	Se6—Re2—Re2^{vi}	55.31 (5)
Se5^{i}—Re1—Re1^{ii}	99.98 (6)	Se6^{v}—Re2—Re2^{vi}	100.10 (6)
Se5^{ii}—Re1—Re1^{ii}	53.66 (5)	Se6^{vi}—Re2—Re2^{vi}	53.80 (4)
Se4—Re1—Re1^{ii}	140.90 (7)	Se3^{iii}—Re2—Re2^{vi}	141.56 (7)
Se4^{iii}—Re1—Re1^{ii}	91.36 (4)	Se3—Re2—Re2^{vi}	91.68 (4)
Se4^{iv}—Re1—Re1^{ii}	135.78 (4)	Se3^{vii}—Re2—Re2^{vi}	135.41 (4)
Se5—Re1—Re1^{i}	55.31 (4)	Re2^{v}—Re2—Re2^{vi}	70.53 (4)
Se5^{i}—Re1—Re1^{i}	53.66 (5)	Re2^{viii}—Se3—Re2	81.52 (6)
Se5^{ii}—Re1—Re1^{i}	99.98 (6)	Re2^{viii}—Se3—Re2^{vii}	98.89 (6)
Se4—Re1—Re1^{i}	91.36 (4)	Re2—Se3—Re2^{vii}	98.89 (6)
Se4^{iii}—Re1—Re1^{i}	140.89 (7)	Re1—Se4—Re1^{viii}	81.37 (6)
Se4^{iv}—Re1—Re1^{i}	135.78 (4)	Re1—Se4—Re1^{iv}	98.73 (6)
Re1^{ii}—Re1—Re1^{i}	70.48 (4)	Re1^{viii}—Se4—Re1^{iv}	98.73 (6)
Se6—Re2—Se6^{v}	109.11 (6)	Re1—Se5—Re1^{i}	71.04 (6)
Se6—Re2—Se6^{vi}	109.11 (6)	Re1—Se5—Re1^{ii}	71.04 (6)
Se6^{v}—Re2—Se6^{vi}	83.12 (7)	Re1^{i}—Se5—Re1^{ii}	83.16 (7)
Se6—Re2—Se3^{iii}	86.40 (6)	Re2—Se6—Re2^{v}	70.89 (6)
Se6^{v}—Re2—Se3^{iii}	95.47 (5)	Re2—Se6—Re2^{vi}	70.89 (6)
Se6^{vi}—Re2—Se3^{iii}	164.06 (7)	Re2^{v}—Se6—Re2^{vi}	83.12 (7)

Symmetry codes: (i) -*x*+1, -*y*+1, -*z*+1; (ii) -*x*+1, -*y*+2, -*z*+1; (iii) *x*, *y*+1, *z*; (iv) -*x*, -*y*+1, -*z*+1; (v) -*x*+2, -*y*+2, -*z*+2; (vi) -*x*+2, -*y*+1, -*z*+2; (vii) -*x*+1, -*y*+1, -*z*+2; (viii) *x*, *y*-1, *z*.

[1] G. Kresse, J. Furthmüller, *Comput. Mater. Sci.* **1996**, 6, 15.
[2] G. Kresse, D. Joubert, *Phys. Rev. B* **1999**, 59, 1758.
[3] J. P. Perdew, K. Burke, M. Ernzerhof, *Phys. Rev. Lett.* **1996**, 77, 3865.
[4] G. Henkelman, B. P. Uberuaga, H. Jónsson, *J. Chem. Phys.* **2000**, 113, 9901.
